# Anomalous Supradominant Left Circumflex Artery with Rare, Rudimentary Left Anterior Descending Artery

**DOI:** 10.7759/cureus.3189

**Published:** 2018-08-23

**Authors:** Ghulam Abbas Shaikh, Ameema Asad, Syed Saadan Ahmed

**Affiliations:** 1 Department of Cardiology, Dr. Ruth K. M. Pfau Civil Hospital, Karachi, PAK; 2 Dow Medical College, Dow University of Health Sciences, Karachi, PAK

**Keywords:** coronary arteries, angina, coronary artery anomaly

## Abstract

Anomalies pertaining to the coronary vasculature are rare and generally asymptomatic, however, they can pose life-threatening risks in the form of sudden cardiac arrests or myocardial ischemia. We present a rare anomaly of a supradominant left circumflex artery (LCX) and a rudimentary left anterior descending artery (LAD). The patient presented in our outpatient department (OPD) with complaints of occasional chest pain and dyspnea on exertion. An exercise tolerance test (ETT) was carried out, which was inconclusive and an electrocardiogram (EKG) revealed a sinus rhythm with a nonspecific ST segment. Due to these findings, an angiography was performed via the trans-radial route using a TIG 5 French catheter (Terumo Medical Corporation, NJ, US). Coronary angiography revealed codominance because of the presence of a rudimentary LAD, a normal right coronary artery (RCA), and an extremely large LCX. As there was no significant stenosis or atherosclerosis in the coronary arteries, pharmacological treatment was chosen. The patient was discharged in a medically stable condition with a routine follow-up planned after one month. The identification of this supradominant LCX is crucial for diagnosis in possible future circumstances of percutaneous coronary intervention or coronary artery bypass grafting operations, ultimately improving the success rate of invasive cardiac therapies.

## Introduction

The concept of coronary dominance is established by the coronary artery that supplies the posterior descending artery (PDA) and, consequently, determines coronary “dominance.” In approximately 70% of the population, the PDA originates from the right coronary artery; it is codominant in 20%, meaning both the right coronary artery (RCA) and left circumflex artery (LCX) feed the PDA; and 10% are left dominant, meaning the LCX alone supplies the PDA. A vessel is considered supradominant when it is extremely large and supplies the region that is normally supplied by another vessel [1].

The usual anatomical variant found in the general population comprises the left main coronary artery (LMCA) typically arising from the left sinus of Valsalva, coursing between the main pulmonary artery and the left auricle before entering the coronary sulcus. The LMCA typically does not have significant branches of its own but quickly bifurcates into the left anterior descending artery (LAD) and the circumflex coronary artery (LCX) [2]. Normally, in most people, the LAD remains the most dominant vessel and supplies about 2/3rd of the heart. It is very rare that the LAD remains rudimentary. In this case report, we present a case of a rare anomaly of a rudimentary LAD occurring with a supradominant LCX.

## Case presentation

A 45-year-old man presented in the outpatient department of a tertiary care hospital with complaints of exertional dyspnea and occasional heaviness in the chest for the past two months. The chest pain described by the patient was unrelated to a specific time of the day. He was a known case of hypertension and well-controlled type II diabetes mellitus with no history of previous hospitalizations. The vitals were well-defined in the accepted range, with blood pressure measuring 130/85, body temperature of 98.6 F, a respiratory rate of 12 breaths per minute, and a pulse of 90 beats per minute. There were no positive signs of pallor, clubbing, or nail bed fluctuations on the general physical examination. The S1 and S2 systolic sounds were normal, without the presence of any added sounds. There was no abnormality in the routine biochemical tests. For further evaluation, an EKG was performed, which exhibited a sinus rhythm with nonspecific ST changes. Due to this, an exercise tolerance test (ETT) was performed after consent from the patient. ETT was inconclusive, as the target heart rate was not achieved and the patient was unable to walk more than 4 metabolic equivalents (METS). Subsequently, an angiography was carried out via the trans-radial route using a TIG 5 French catheter. 

**Figure 1:**
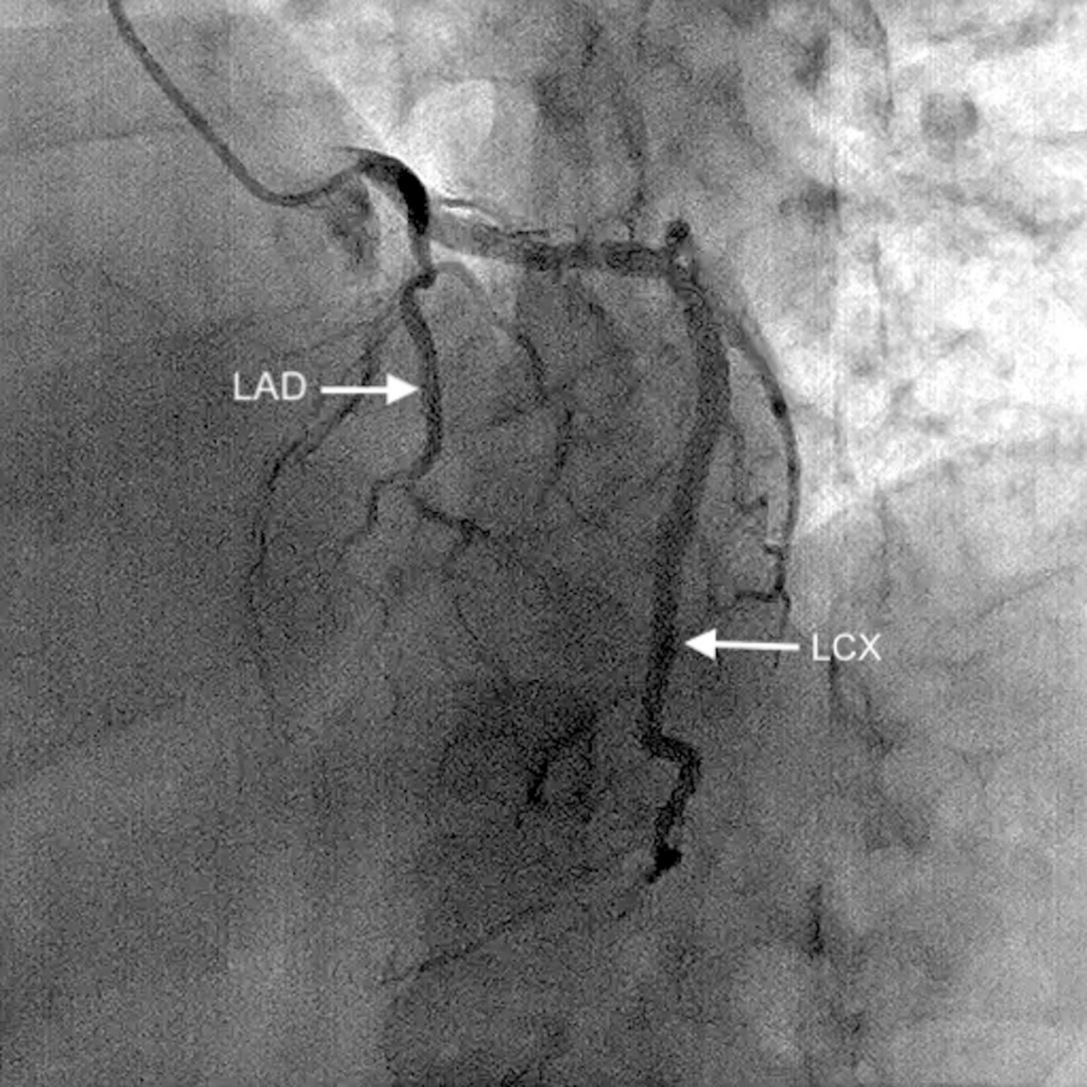
Rudimentary LAD, which is seen to end abruptly. LAD: left anterior descending artery

**Figure 2:**
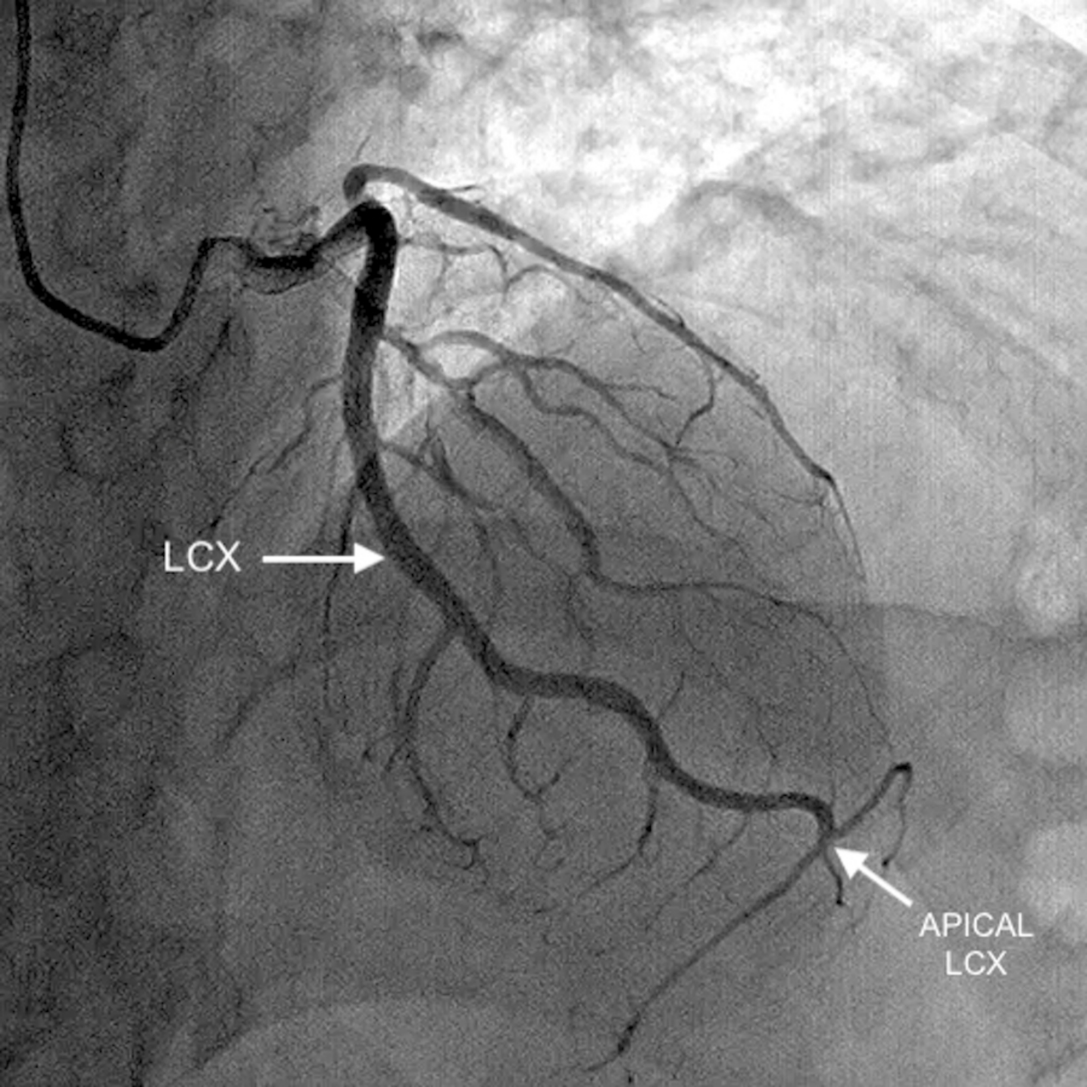
Extremely large LCX is seen coursing toward the apex with the apical LCX supplying the territory of the LAD. LCX: left circumflex artery; LAD: left anterior descending artery

**Figure 3:**
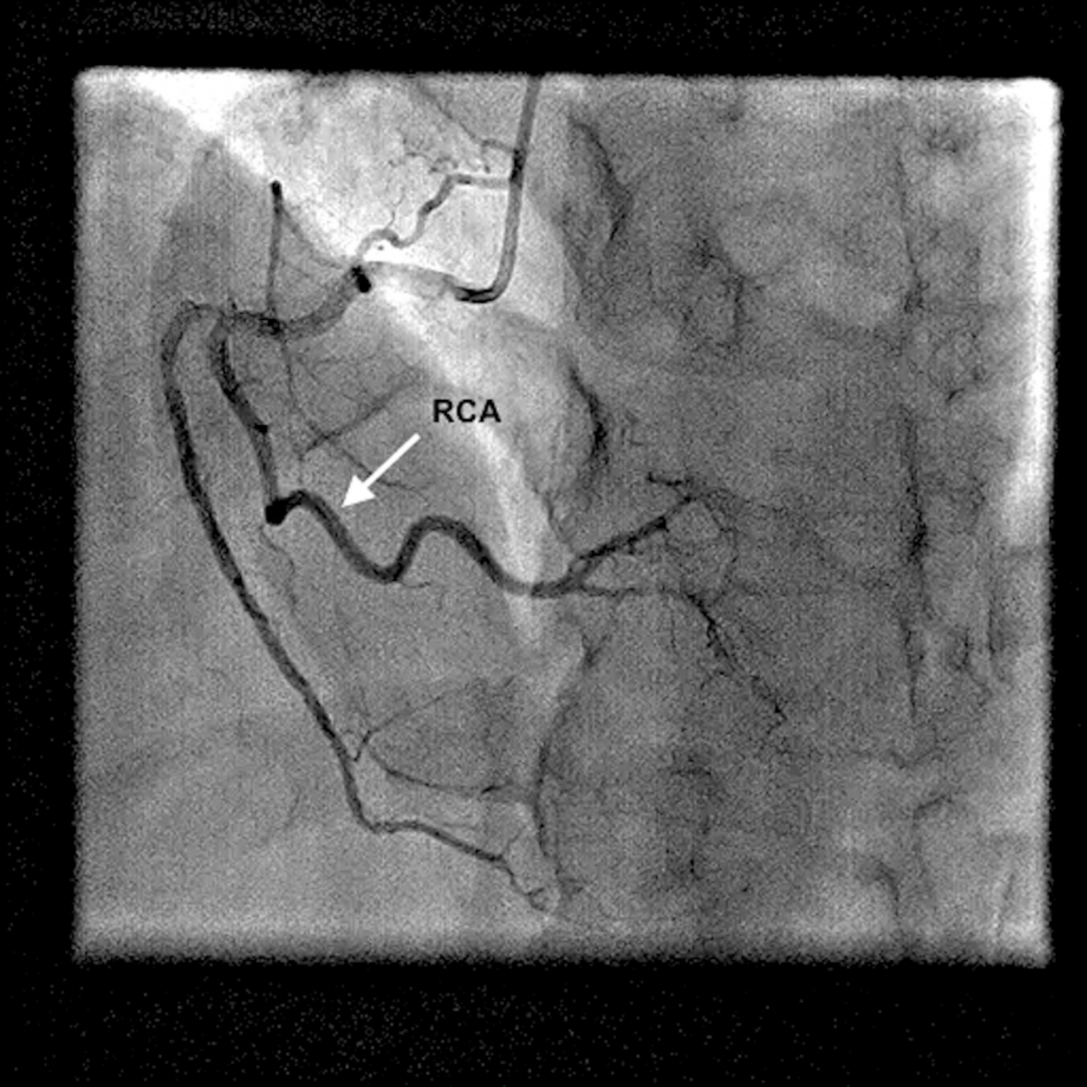
Normal-sized RCA is seen supplying the PDA. RCA: right coronary artery; PDA: posterior descending artery

Coronary angiography indicated co-dominance, along with the presence of a rudimentary LAD, which abruptly tapered at the mid-level before reaching the left ventricular apex (Figure 1) (Video 1). The LCX was enormous and coursed toward the apex, in the coronary sulcus, onto the diaphragmatic cardiac surface and distally rather than ending before reaching the posterior interventricular sulcus, thus behaving like the LAD (Figure 2) (Video 2). The right coronary artery (RCA) was also dominant and supplied the PDA (Figure 3) (Video 3). Thus, it was apparent that the LCX was indeed supradominant in its course of supply. There was no hemodynamically significant stenosis and atherosclerosis in the coronary arteries; therefore, the symptoms were treated pharmacologically. The patient was discharged in a medically stable condition with a routine follow-up planned after one month.

**Video 1:** Coronary angiography video for Figure 1.

**Video 2:** Coronary angiography video for Figure 2.

**Video 3:** Coronary angiography video for Figure 3.

## Discussion

A coronary artery anomaly (CAA) is defined as an abnormality in the origin, course, or distribution of the blood supply of an artery that may or may not be associated with clinically significant symptoms. The incidence of CAAs varies widely in the literature; which can be a reflection of referral bias and variability in the definitions of “anomalous” and “normal variant”. With reference to this, they are believed to affect around 1% of the general population, ranging from 3%-5.6% in studies on patients undergoing coronary angiography, and in approximately 1% of routine autopsy [3]. CAAs can be clinically classified as benign with an incidence of 81% and malignant with an incidence of 29%[4]. In a study conducted at the Cleveland Clinic Foundation, the majority of CAAs discovered did not result in any signs, symptoms, or complications, and they usually were discovered as incidental findings at the time of catheterization [4]. Consequently, it is reiterated from the literature that CAAs are usually encountered incidentally during a coronary angiography.

The identification of CAAs through cardiovascular computed tomography (CT) images is important because the suspicion that a patient’s problems may be the result of coronary anomalies remains a crucial challenge in diagnosis [5]. In nearly 15% of patients with CAAs, myocardial ischemia can develop in the absence of atherosclerosis [3]. This is reinforced by Maron et al. who reported that 19% of deaths in athletes is attributable to CAAs, making them the second-most common cause of sudden death in young athletes [6].

In our patient, the abnormally large LCX originated from the left main coronary artery (LMCA) and supplied the lateral wall of the left ventricle, the apex (which is usually supplied by the LAD), and variable portions of the inferior wall through the obtuse marginal and posterolateral branches, respectively. It was also found to supply a portion of the inferior wall and the atrioventricular node. However, the LAD was rudimentary and might constitute a false identification of an occluded or even absent LAD. Furthermore, the LAD is the most commonly occluded coronary artery [7]; the presence of an already short LAD along with this risk factor increases the chances of myocardial ischemia. The identification of this supradominant LCX is critical for future diagnoses in any given circumstances of percutaneous coronary interventions or coronary artery bypass grafting operations (CABG), as well as the correct placement of the graft, ultimately improving the success rate of invasive cardiac therapies.

## Conclusions

The supradominant nature of LCX with the unusually small LAD, which, in usual cases, is the most significant coronary artery, implies an evident risk for the future in the case of atherosclerosis or occlusion, which can lead to the complete shut-off of large areas of the myocardium, causing catastrophic complications. Thus, an improper recognition of unusual coronary anatomy may lead to incorrect diagnosis or treatment, both in the catheterization laboratory and at the surgical suite.
